# Supplementary material for: Surface superconductivity in the topological Weyl semimetal t-PtBi2
Source: Nat Commun. 2024 Nov 15;15:9895. doi: 10.1038/s41467-024-54389-6 (PMC11568133; doi:10.1038/s41467-024-54389-6)
Supplement: Supplementary file 1 — Supplementary Information [file 41467_2024_54389_MOESM1_ESM.pdf]

Supplementary information:

Surface superconductivity in the topological Weyl semimetal t-$PtBi_2$

Sebastian Schimmel[1,2,*], Yanina Fasano[2,3], Sven Hoffmann[1,2], Julia Besproswanny[1,2], Laura Teresa Corredor Bohorquez[2], Joaquín Puig[2,3], Bat-Chen Elshalem[4], Beena Kalisky[4], Grigory Shipunov[2,†], Danny Baumann[2], Saicharan Aswartham[2], Bernd Büchner[2,5], Christian Hess[1,2,*]

[1] *Fakultät für Mathematik und Naturwissenschaften, Bergische Universität Wuppertal, 42097 Wuppertal, Germany*

[2] *Leibniz-Institute for Solid State and Materials Research (IFW-Dresden), Helmholtzstraße 20, 01069 Dresden, Germany*

[3] *Instituto de Nanociencia y Nanotecnología and Instituto Balseiro, CNEA – CONICET and Universidad Nacional de Cuyo, Centro Atómico Bariloche, Avenida Bustillo 9500, 8400 Bariloche, Argentina*

[4] *Department of Physics and Institute of Nanotechnology and Advanced Materials, Bar-Ilan University, Ramat-Gan 5290002, Israel.*

[5] *Institute of Solid State and Materials Physics and Würzburg-Dresden Cluster of Excellence ct.qmat, Technische Universität Dresden, 01062 Dresden, Germany*

[*] *Corresponding authors*

[†] *Present address: Institute of Physics, University of Amsterdam, 1098 XH Amsterdam, The Netherlands*

Content

A. Scanning SQUID on t-$PtBi_2$ 2

B. Additional fits of the superconducting gap 3

C. Supplementary topographic and spectroscopic information to main text Figure 2 4

D. Spectroscopic and topographic information on additional t-$PtBi_2$ samples 10

E. Specific heat of t-$PtBi_2$ 15

A. Scanning SQUID on t-$PtBi_2$

In Supplementary Figure 1 results of scanning SQUID measurements performed at 6.4 K on a crystal from the same batch is shown. Panels (c, d) show a weak but measurable diamagnetic signal near the surface of the investigated sample. The observed signals are comparable in magnitude to the diamagnetic signal of a 2D superconductor (see https://www.nature.com/articles/nphys2079). Note, however, that no Pearl vortices were observed at these temperatures by scanning SQUID, and no critical temperature was found in these preliminary measurements.

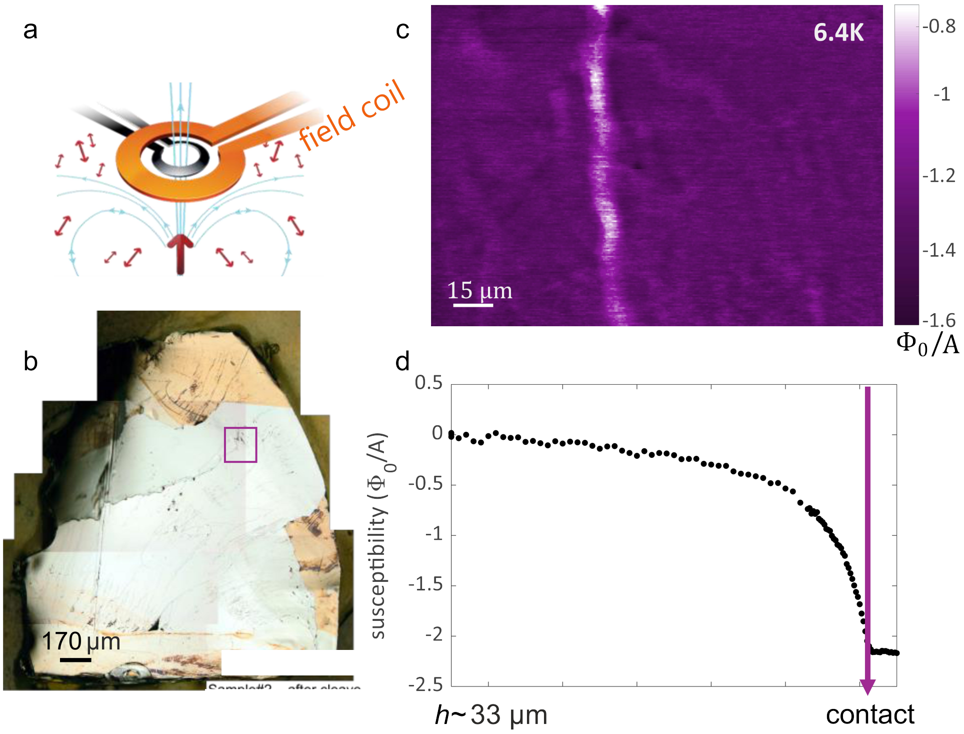

Supplementary Figure 1: (a) Illustration of scanning SQUID measurement. The sensor captures field lines that are generated by the sample. The outer loop, the field coil, applies a local magnetic field to measure susceptibility as a function of location. (b) Optical picture of the measured sample. (c) Local susceptibility map of the area marked by a purple square in b, taken at 6.4 K. The susceptibility is measured in units of Φ_0, normalized by the current in the field coil. (d) Susceptibility at 6.8 K as a function of spacing between the sensor and the sample. The signal is strongest at the contact point (marked by an arrow).

B. Additional fits of the superconducting gap

The following Supplementary Figures 2 and 3 show examples of additional fitting attempts for the data shown in Fig. 2(b). Supplementary Figure 2 presents fit attempts (Methods Equation (1)) with two *s*-wave gaps with a single (a) and two independent Γ values (b). Supplementary Figure 3 shows a fit with a nodal order parameter (Methods Equation (2)). In all cases, the leading gap is of similar order. Note that the data have been normalized.

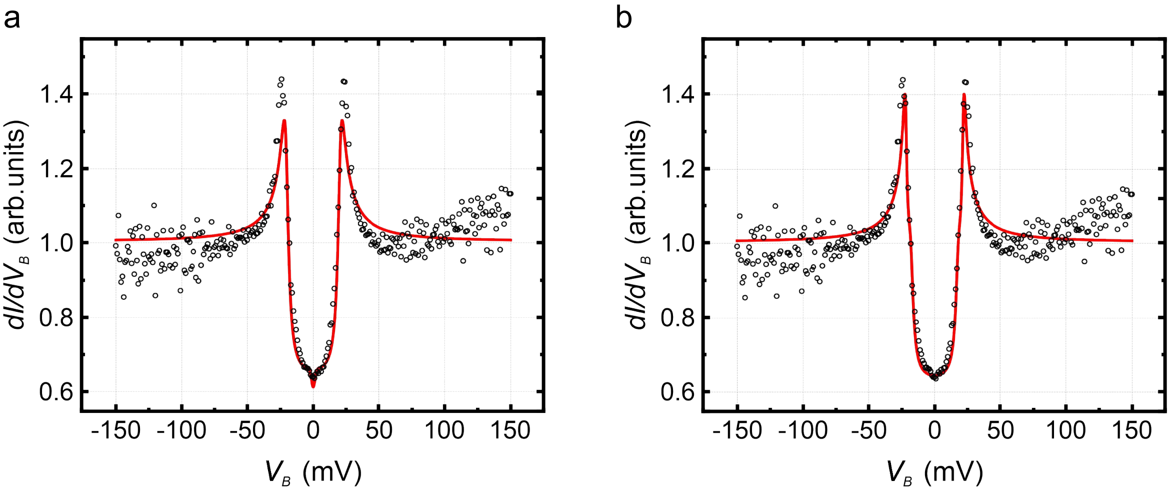

$\Delta_1 = 20.3276$ meV
$\Delta_2 = 1.2187$ meV
$\Gamma = 2.4292$ meV
$D = 0.5827$
$R^2 = 0.8373$

$\Delta_1 = 21.7670$ meV
$\Delta_2 = 17.5591$ meV
$\Gamma_1 = 1.2496$ meV
$\Gamma_2 = 2.1978$ meV
$D = 0.6086$
$R^2 = 0.8544$

Supplementary Figure 2: The red curves show the results of fit attempts of the data in Fig. 2(b) with two *s*-wave gaps with a single (a) and two independent Γ values (b).

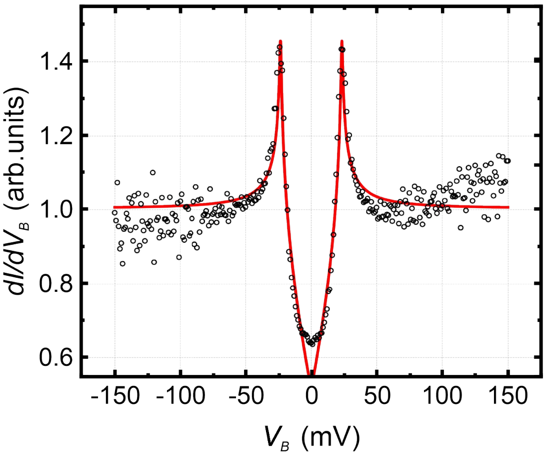

$\Delta = 23.3523$ meV; $\Gamma = 0.5235$ meV; $D = 0.4884$; $R^2 = 0.8437$

Supplementary Figure 3: The red curve shows the result of a fit of the data in Fig. 2(b) using a nodal order parameter.

C. Supplementary topographic and spectroscopic information to main text Figure 2

Supplementary Figure 4 shows additional information to the main text Fig. 2(a). In Supplementary Figure 4(a) the topography of the 150x150 nm^2 field of view (FOV) is presented, where the gapped zero-field spectrum (see Fig. 2(a) in the main text or Supplementary Figure 4(c)) was measured. In agreement with the representative high resolution topography of the type B surface shown in the main text Fig. 1(d), the surface appears atomically flat and homogeneous. The homogeneity is only interrupted by a few atomic defects of a density < 0.5 % per unit cell. The inserted green square marks the area shown in Supplementary Figure 4(b), in which the typical atomic corrugation of the type B surface can be seen more clearly. The appearance of the atomic corrugation is dominated by bright structures that remind of a lattice composed of honeycombs that are distorted into triangles. Besides the ideal structure a characteristic defect of the type B surface can be found at the lower left of the centre of this image (red circle). The location were the spectrum of Supplementary Figure 4(c) was measured is indicated by a black circle with a dot at its centre.

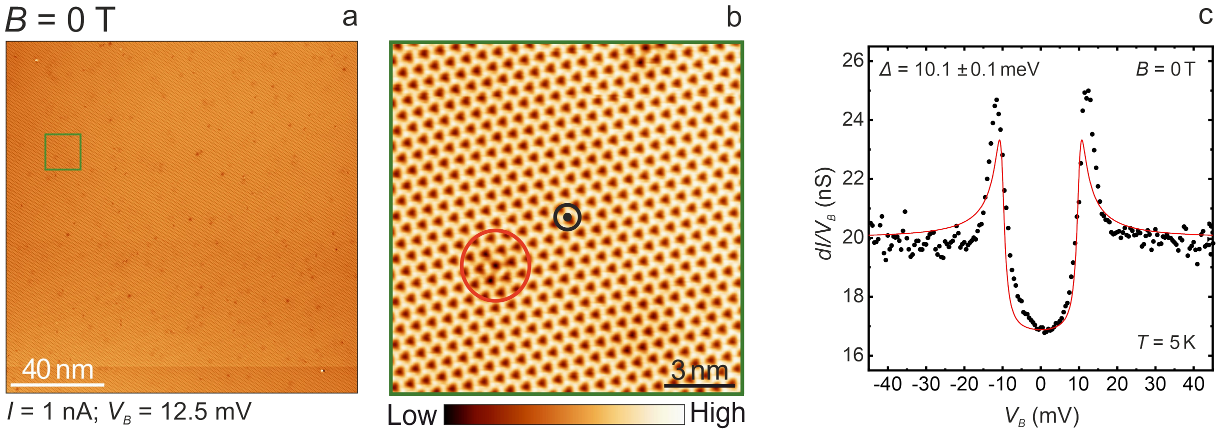

Supplementary Figure 4: Topography and spectroscopy of sample #1 in zero-field. The topography of the total field of view and the digital zoom of the area highlighted by the the green square are presented in (a) and (b) respectively. The spectrum measured at the position marked by the black circle with a dot at its centre is presented in (c). The Dynes fit (Eq. (1)) of the data is presented in red.

The overview topography where the spectrum that exhibits the largest gap of this study was acquired (Fig. 2(b) in the main text or Supplementary Figure 5c)) is presented in Supplementary Figure 5(a). Even though measured 3 weeks posterior to the zero-field data (Supplementary Figure 4), the surface is atomically clean and the amount of atomic defects corresponds to that of topography Supplementary Figure 4(a). At the top of the FOV a wrinkle that appears as a bright linear feature in the image disturbs the overall flatness of the surface. The atomic corrugation is presented in Supplementary Figure 5(b) that shows the digitally enlarged area

marked by a green square in Supplementary Figure 5(a). In comparison to Supplementary Figure 4(b), the appearance of the atomic corrugation of the type B surface is slightly altered (presumably due to a tip change), still featuring the characteristics of the type B surface. A black circle with a dot at its centre indicates the position in Supplementary Figure 5(b) where the spectrum Supplementary Figure 5(c) was measured.

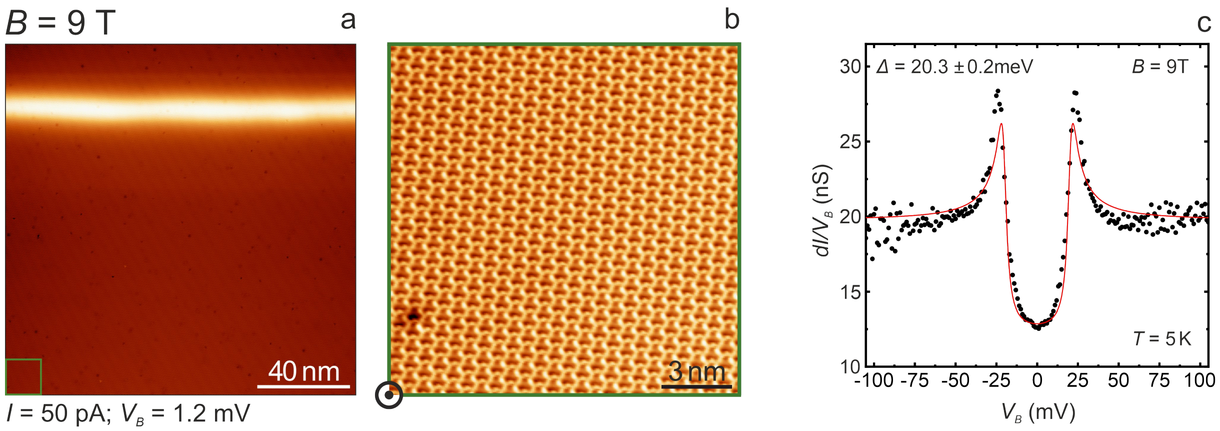

Supplementary Figure 5: Topography and spectroscopy of sample #1 in $B = 9$ T. The topography of the total field of view and the digital zoom of the area highlighted by the the green square are presented in (a) and (b) respectively. The spectrum measured at the position marked by the black circle with a dot at its centre is presented in (c). The Dynes fit (Eq. (1)) of the data is presented in red.

The evolution of the quasiparticle excitation spectrum with applied magnetic field (Fig. 2(c) in main text) is based on the topographic and spectroscopic data presented in Supplementary Figure 6. All overview topographies Supplementary Figure 6(a, d, g, j, m) testify the atomic flatness and homogeneity of the surface according to Supplementary Figure 4(a). The magnified area Supplementary Figure 6(b) which is marked by a green square in Supplementary Figure 6(a) shows the representative atomic corrugation. The location where the 3 T point spectrum (Supplementary Figure 6(c)) was measured is indicated by a black circle with a dot at its centre. To acquire the spectroscopic data in higher B-fields, grid spectroscopic measurements were performed. The positions of the single spectra are marked in the corresponding topographies (Supplementary Figure 6(d, g, j, m)) and the measured associated spectra are plotted in Supplementary Figure 6(e, h, k, n). The resulting average spectra are shown in Supplementary Figure 6(f, i, l, o) respectively and are presented in Fig. 2(c) of the main text. A closer inspection of the data yields that the magnitude of the superconducting gaps varies only slightly within the areas under investigation.

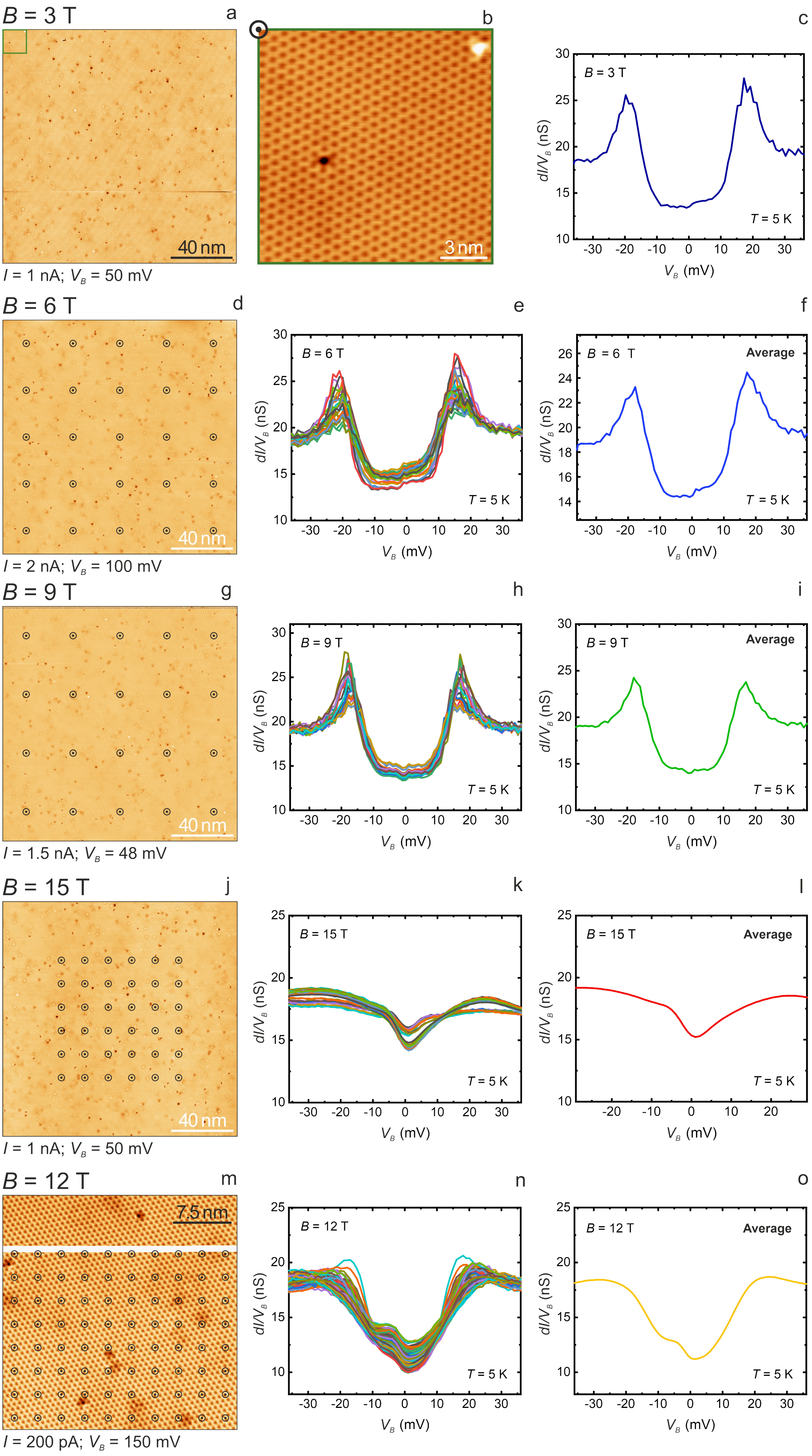

Supplementary Figure 6: Topography and spectroscopy of sample #1 in varying *B*-fields. Details are given in the text. The colours of the spectroscopic curves are used solely for distinction.

Supplementary Figure 7 shows the composition of the FOVs of Supplementary Figure 4, 5 and 6(a, d, g, j ,m) in which the data from zero field up to 15 T was acquired within a time of 3 weeks. Note, that the largest measured gap shown in Fig. 2b/Supplementary Figure 5(c), recorded at the location which is marked by a blue circle in Supplementary Figure 7, was not reproduced in the grid spectroscopy measurement performed on the same surface (Supplementary Figure 6(d, e, f) and Supplementary Figure 6(g, h, i)).

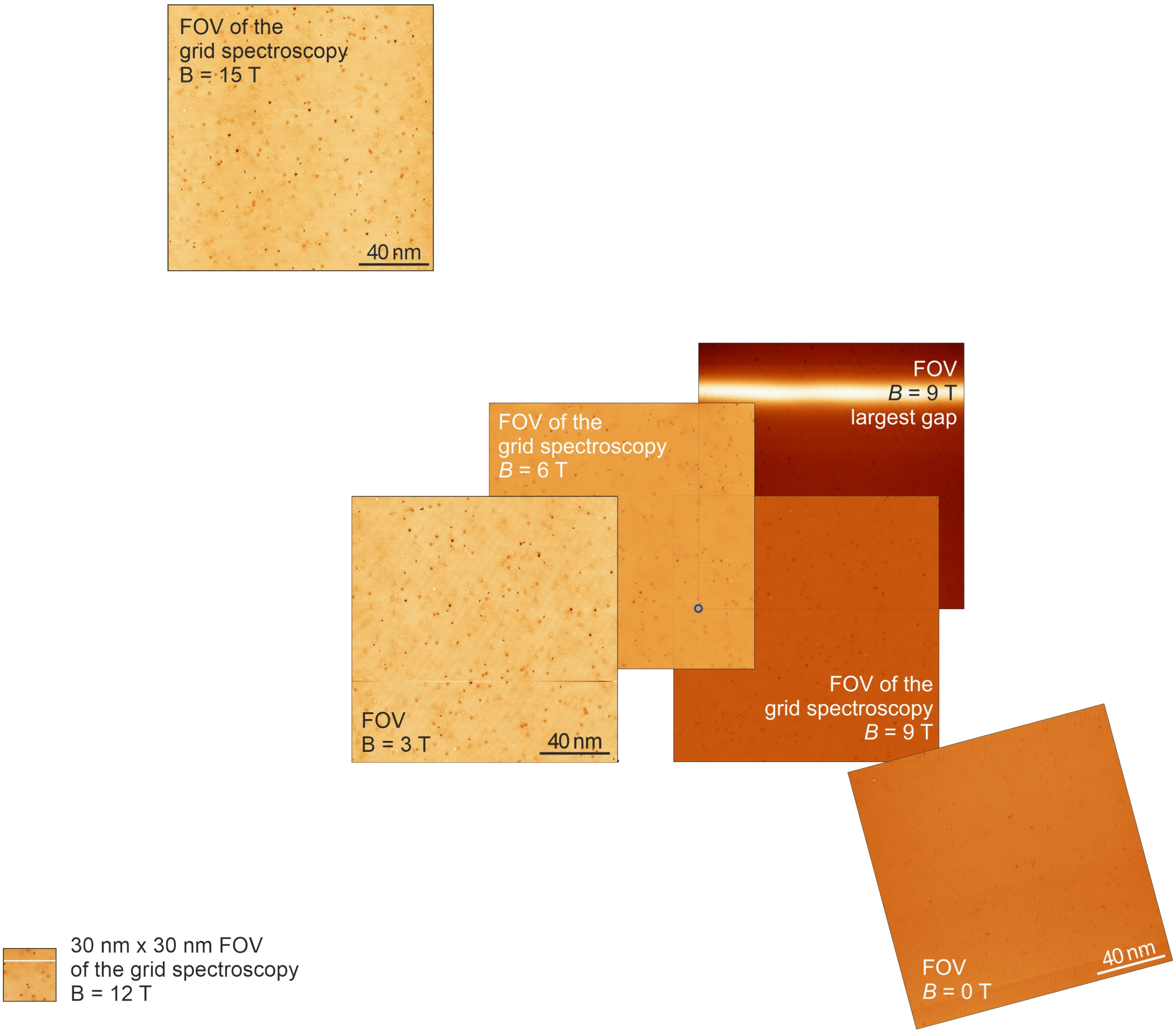

Supplementary Figure 7: Composed field of views of measurements on sample #1 in *B*-fields. The different leading colours of the separately measured and collectively presented areas only serve the purpose to distinguish between them.

In order to visualise possible vortices, variations of the local density of states were imaged by means of a differential conductance map (dI/dV_B-map) measured at $B = 9$ T and $T = 5$ K in the type B surface of t-$PtBi_2$ sample #1 (Supplementary Figure 8(a)). The top left insert shows the tunnel conductance spectrum measured in advance on the area under investigation. In Supplementary Figure 8(b) the simultaneously acquired atomic-resolution topography is presented. Note, that no signatures of local variations of conductance as expected when nucleating three dimensional vortices are observed even though the map registers the atomic corrugation. The schematic vortex lattice in the bottom right insert in Supplementary Figure 8(a) demonstrates the Abrikosov lattice expected for $B = 9$ T with a lattice spacing of 16 nm.

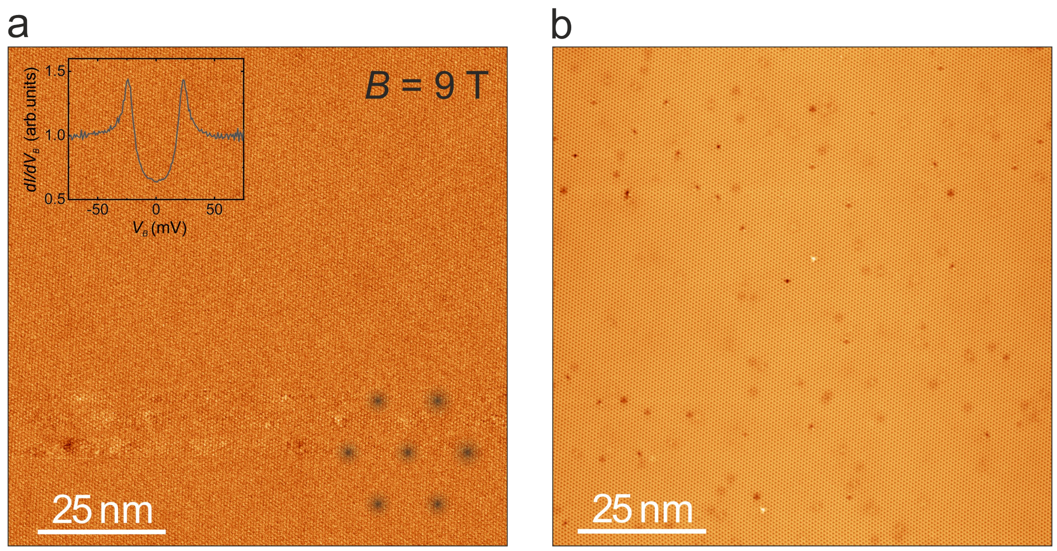

Supplementary Figure 8: dI/dV_B-map in B-field and inside the superconducting gap. The dI/dV_B-map (a) was acquired in parallel to the topography scan (b) under the stabilisation conditions $I = 100$ pA and $V_B = 1.2$ mV and in a magnetic field of $B = 9$ T. The inset in the upper left corner in (a) shows the corresponding spectrum. The inserted dark spots in the lower right corner shows a scheme of vortex lattice corresponding to the field of $B = 9$ T.

Complementary to Fig. 2(d) of the main text, atomic resolution topographies measured at $T = 30$ mK of the samples #10 and #5, which possess the two opposite surface terminations (type A and type B) are presented in Supplementary Figure 9(a) and (c). The corresponding spectra are shown in Supplementary Figure 9(b) for the type A surface and Supplementary Figure 9(c) for the type B surface. The locations where the spectra were taken are indicated by black circles with a dot at its centre.

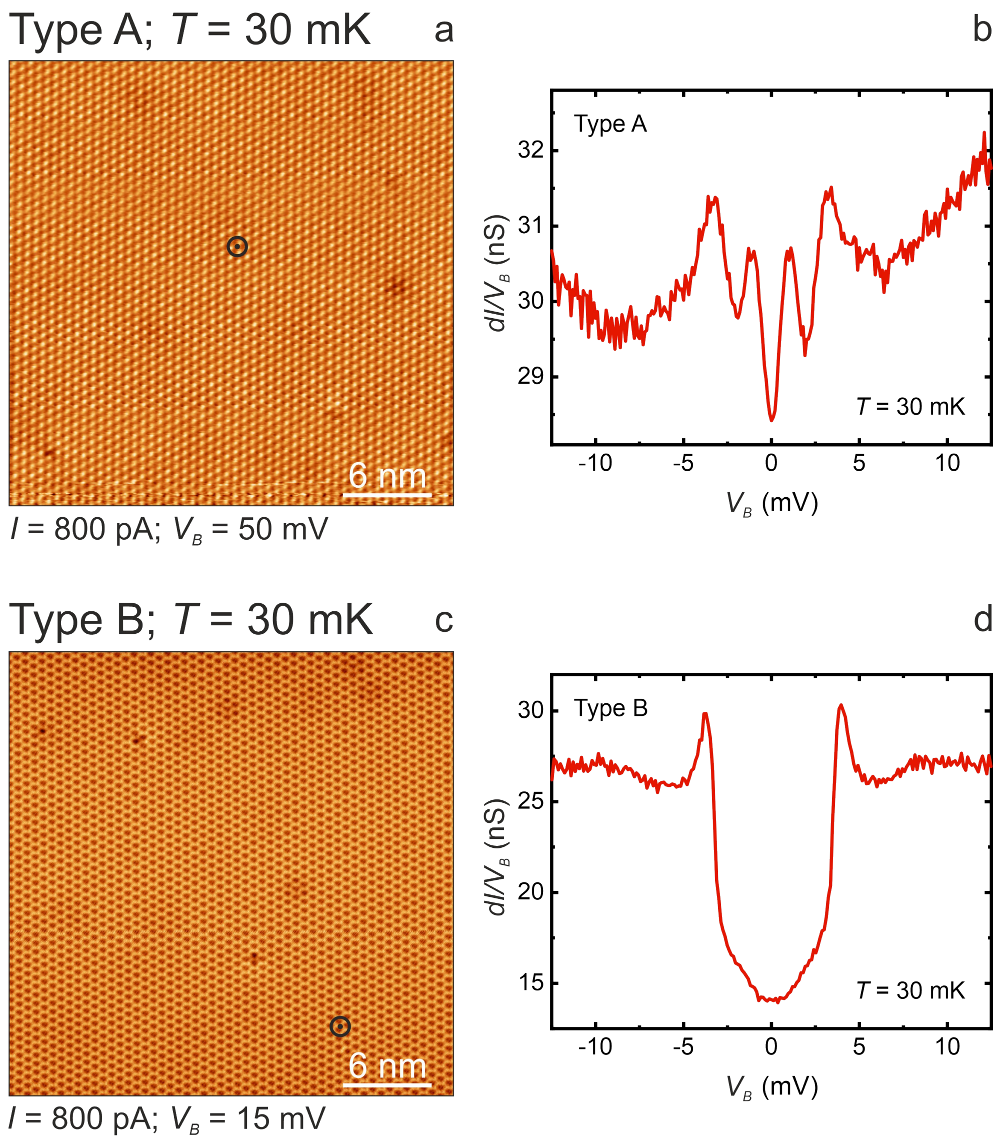

Supplementary Figure 9: Topography and spectroscopy of sample #5 and #10 measured at $T = 30$ mK. The topographies of the investigated areas of a type A and type B surface are presented in (a) and (c) respectively. The corresponding spectra are presented as red curves in (b) and (d).

D. Spectroscopic and topographic information on additional t-$PtBi_2$ samples

Besides the previously discussed, further samples were studied and their spectroscopic information is summarized and jointly presented with chosen earlier mentioned spectra in Supplementary Figure 10. The spectra measured on different samples exhibit a variety of electronic structures at the Fermi level, ranging from nearly featureless metallic, over a reduced zero bias conductance, to well-developed superconducting gap structures characterised by particle hole symmetric coherence peaks.

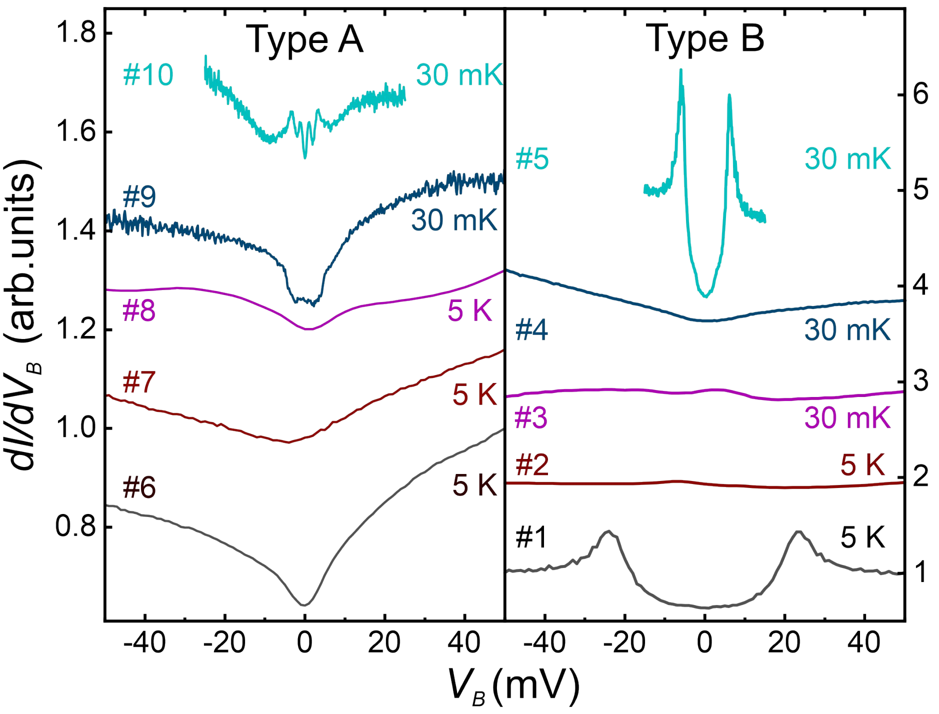

Supplementary Figure 10: Typical normalized tunnel conductance spectra measured in the 10 t-$PtBi_2$ samples studied. Left panel: Data in type A surfaces. Right panel: Data in type B surfaces. Spectra have been shifted vertically and coloured for clarity. Measurements performed at 5 K and 0.03 K.

Complementary topographic and spectroscopic information can be found in Supplementary Figures 11-18. Topographies of the different sample surfaces are listed in the left column (Supplementary Figure 11-18(a, d where applicable)). The point spectroscopic data Supplementary Figure 11-18(b) corresponding to the particular sample is presented on the right of the topography image in the same row. The positions where the spectra were measured in the topographies are marked by black circles with a dot at their centre. Where applicable, averaged spectra are shown in Supplementary Figure 11-18(c).

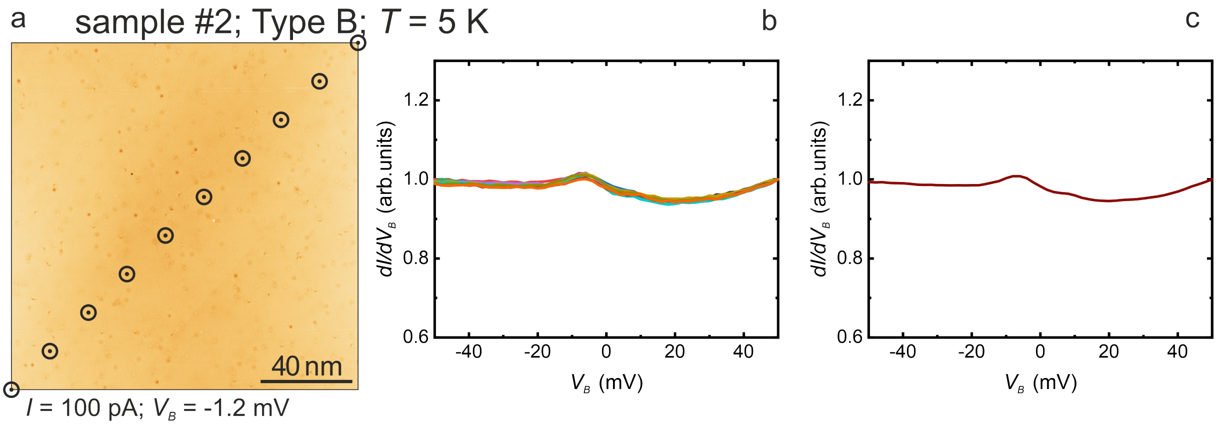

Supplementary Figure 11: Topography and spectra of sample #2 measured at $T = 5$ K. The topography of the area under investigation is presented in (a). All spectra measured along the line of points indicated by black circles in (a) are shown in (b). In (c) the average of the spectra in (b) is shown. The colours of the spectroscopic curves are for visual distinction only.

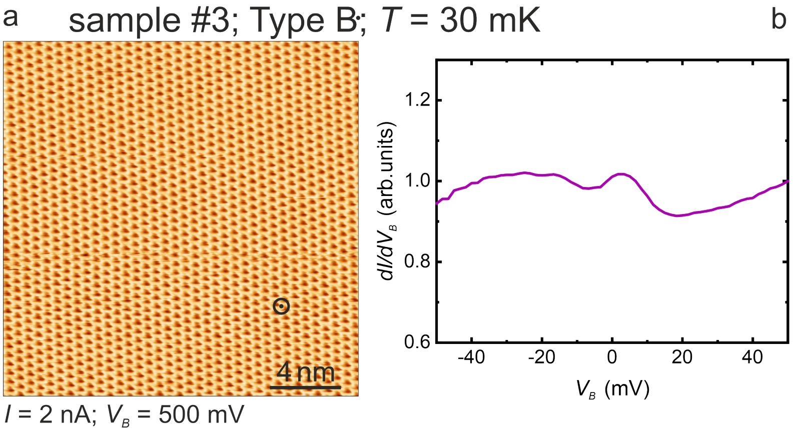

Supplementary Figure 12: Topography and spectroscopy of sample #3 measured at $T = 30$ mK. The topography of the area under investigation is presented in (a). The spectrum measured at the position indicated by a black circle with a dot at its centre in (a) is shown in (b). The colour of the spectroscopic curve is for visual distinction only.

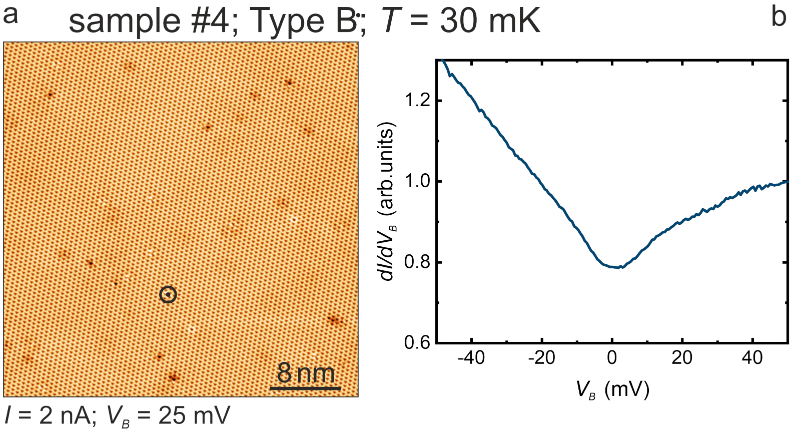

Supplementary Figure 13: Topography and spectroscopy of sample #4 measured at $T = 30$ mK.The topography of the area under investigation is presented in (a). The spectrum measured at the position indicated by a black circle with a dot at its centre in (a) is shown in (b). The colour of the spectroscopic curve is for visual distinction only.

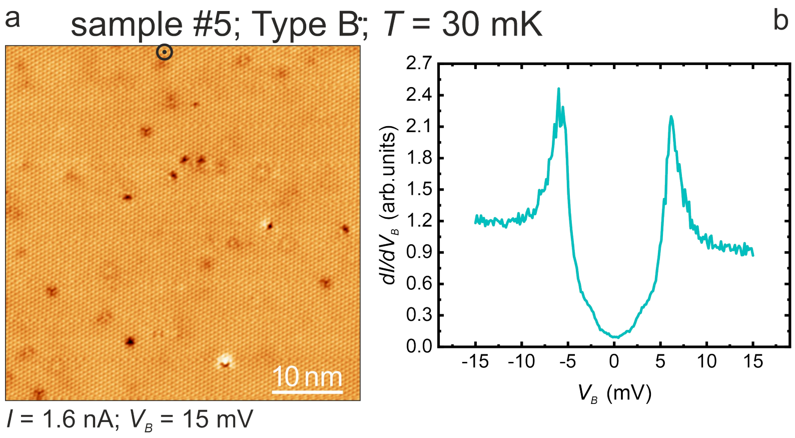

Supplementary Figure 14: Topography and spectroscopy of sample #5 measured at T = 30 mK. The topography of the area under investigation is presented in (a). The spectrum measured at the position indicated by a black circle with a dot at its centre in (a) is shown in (b). The colour of the spectroscopic curve is for visual distinction only.

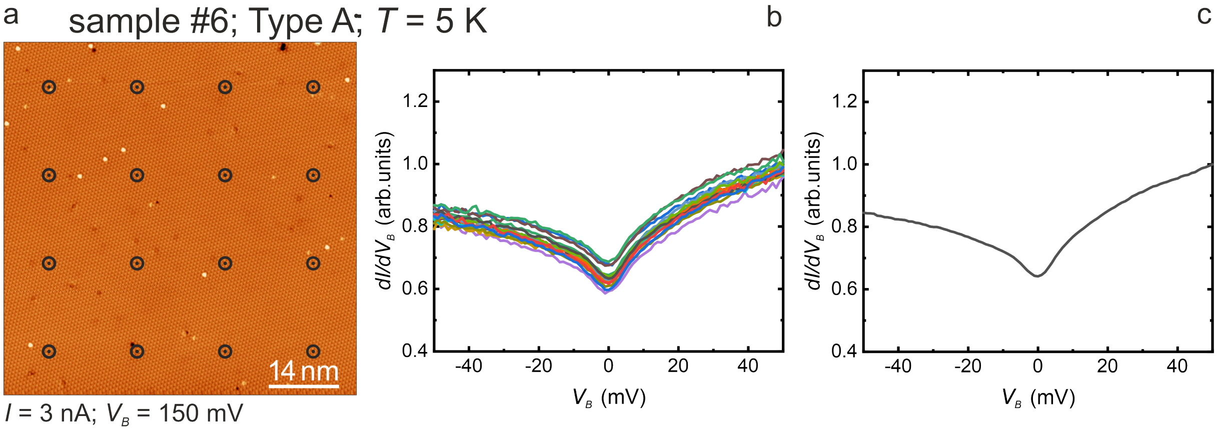

Supplementary Figure 15: Topography and spectra of sample #6 measured at T = 5 K. The topography of the area under investigation is presented in (a). All spectra measured on the grid of points indicated by black circles in (a) are shown in (b). In (c) the average of the spectra in (b) is shown. The colours of the spectroscopic curves are for visual distinction only.

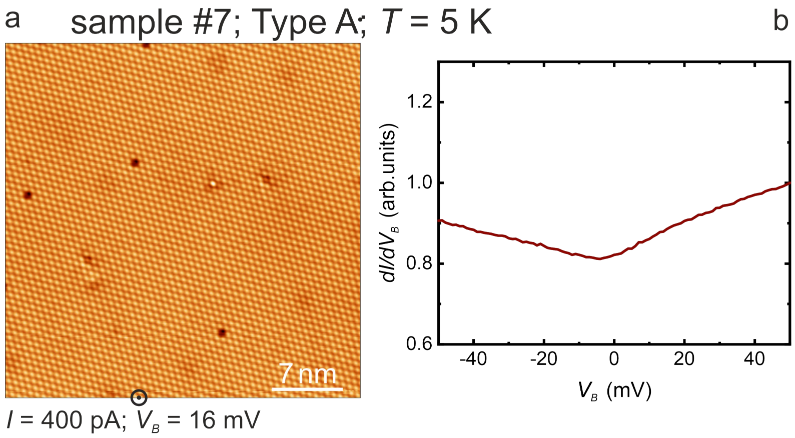

Supplementary Figure 16: Topography and spectroscopy of sample #7 measured at T = 5 K. The topography of the area under investigation is presented in (a). The spectrum measured at the position indicated by a black circle with a dot at its centre in (a) is shown in (b). The colour of the spectroscopic curve is for visual distinction only.

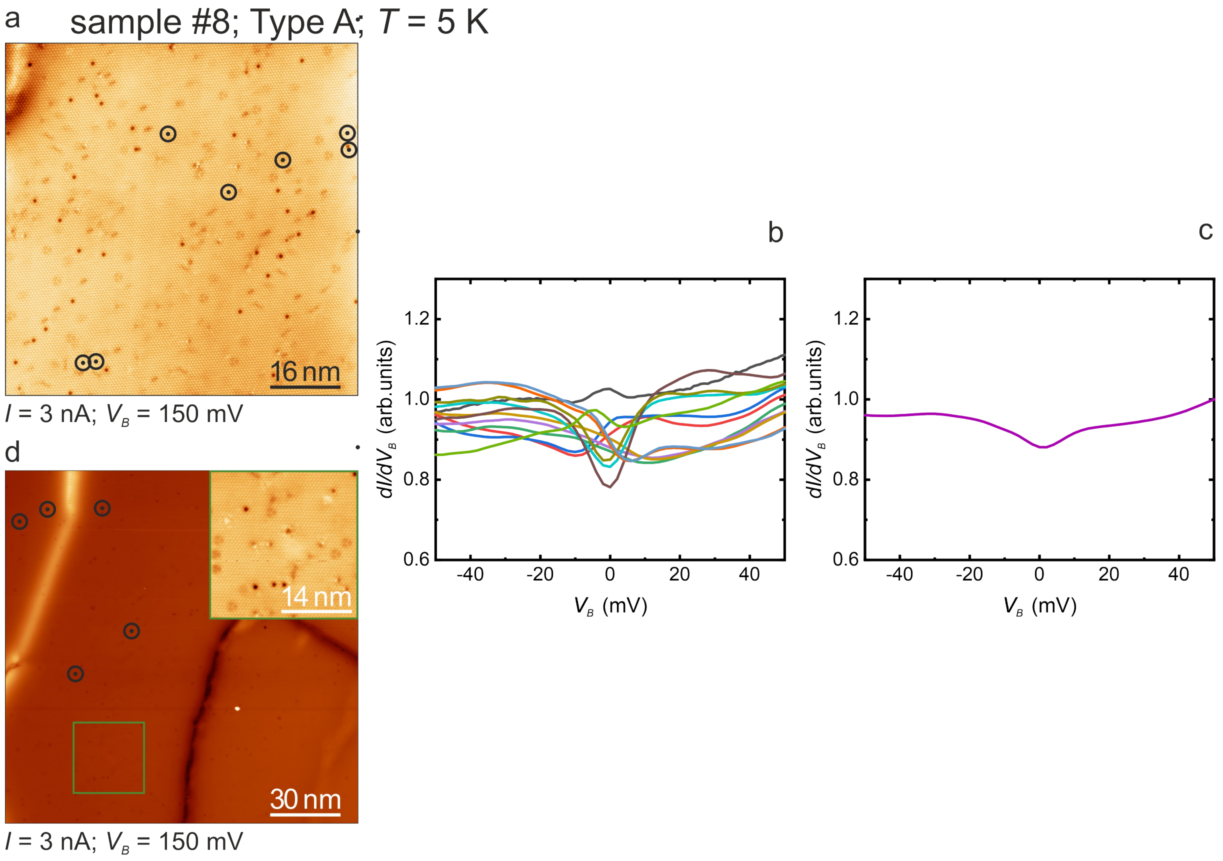

Supplementary Figure 17: Topographies and spectra of sample #8 measured at $T = 5$ K. The topography of the areas under investigation are presented in (a) and (d). The variaty of spectra measured on random points indicated by black circles in (a) and (d) are shown in (b). In (c) the average of the spectra in (b) is shown. The colours of the spectroscopic curves are for visual distinction only.

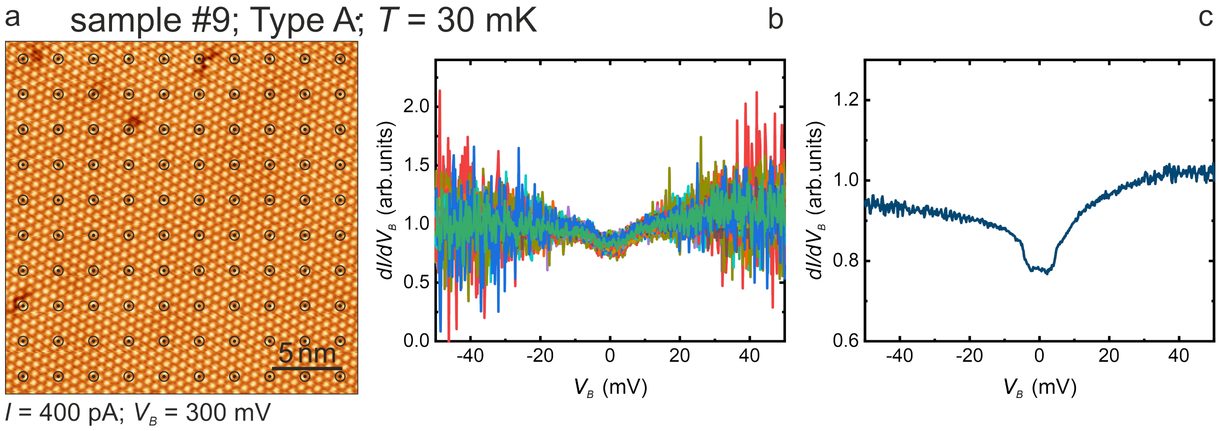

Supplementary Figure 18: Topography and spectra of sample #9 measured at $T = 30$ K. The topography of the area under investigation is presented in (a). All spectra measured on the grid of points indicated by black circles in (a) are shown in (b). In (c) the average of the spectra in (b) is shown. The colours of the spectroscopic curves are for visual distinction only.

As addition to the main text Fig. 3, in Supplementary Figure 19(a) the atomic resolution topography of the FOV of the gap map Supplementary Figure 19(c)/Fig. 3(b) is shown. Presumably due to a tip change, the atomic corrugation could not be resolved with the same quality as in Supplementary Figure 9(c). Nevertheless, the surface type can be doubtlessly recognized by the symmetry and shape of the characteristic atomic defects. The spectra on which the gap map is based (Supplementary Figure 19(b)) were acquired by a grid spectroscopic measurement as indicated by the black circles with a dot at their centre in the topographic image (Supplementary Figure 19(a)).

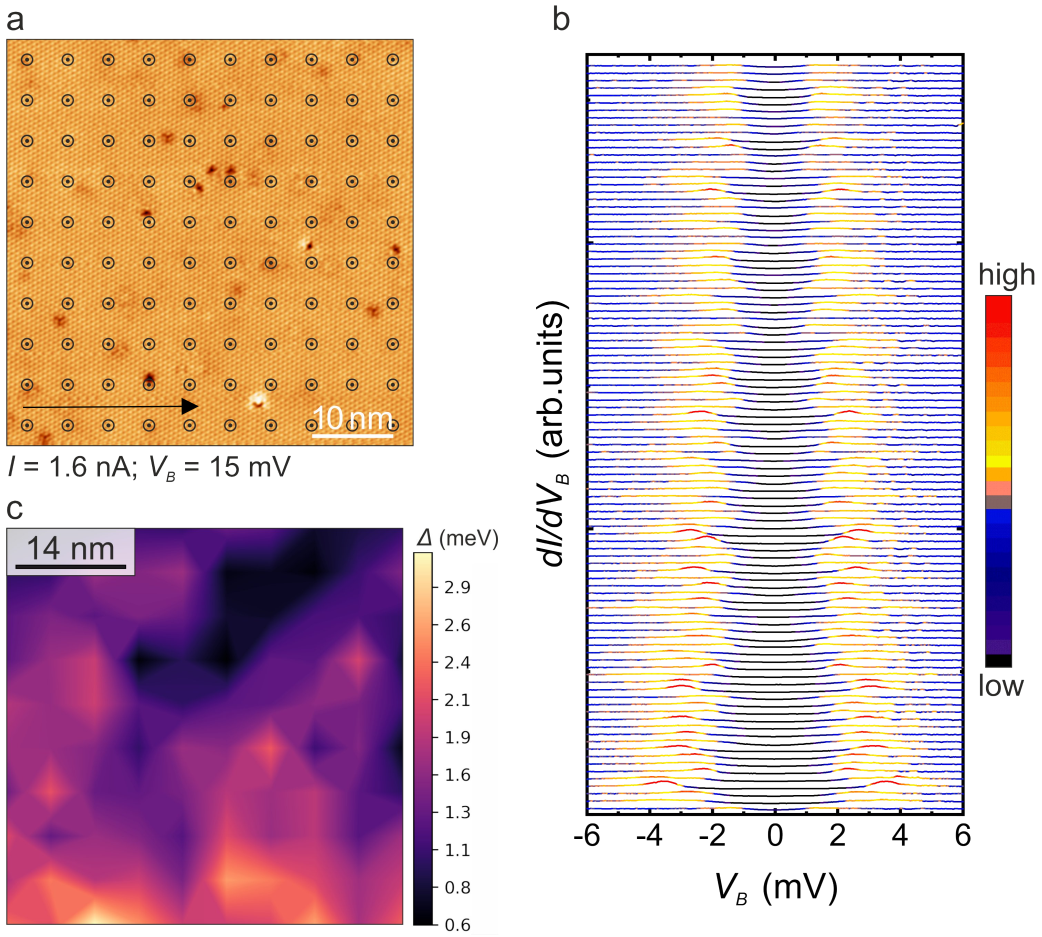

Supplementary Figure 19: Topography, spectroscopy and gap map of sample #5 measured at $T = 30$ mK. The topography of the area under investigation is presented in (a). In (b), from bottom to top the spectra have been registered at the positions indicated in (a), following the order as indicated by the arrow, line by line to the top. Each spectrum is shifted by an offset. The corresponding gap map is visualized in (c).

E. Specific heat of t-$PtBi_2$

In order to probe for signatures of bulk superconductivity, we performed specific heat measurements as a function of temperature down to $T \approx 500$ mK. Supplementary Figure 20 presents the data as a function of temperature in zero field and for finite magnetic fields. The data reveal no sign of any bulk superconducting transition in the measured range, in particular at around 600 mK, where resistivity reports a drop to zero.

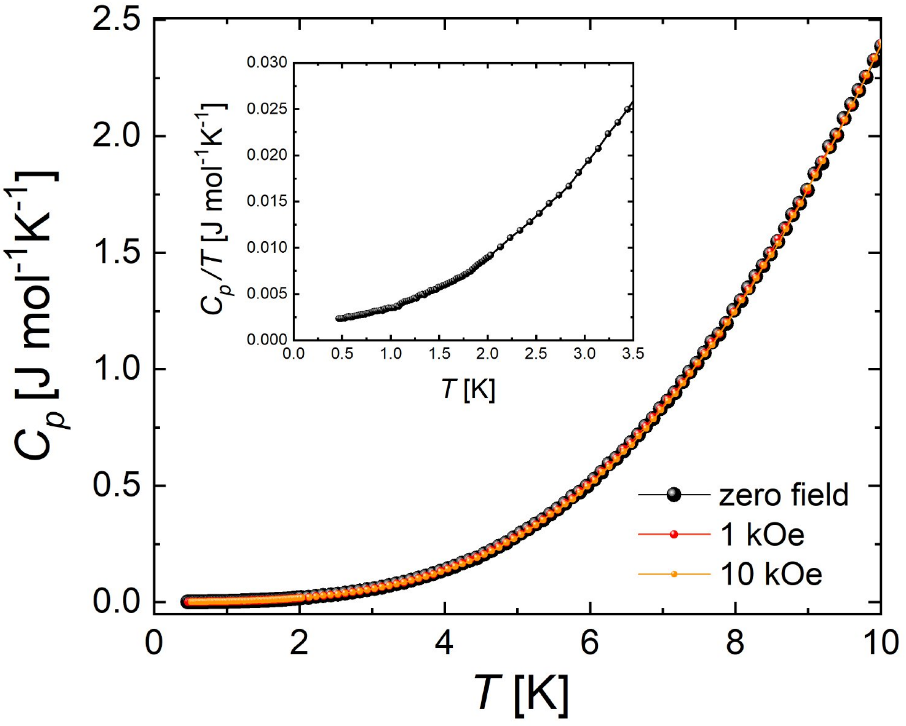

Supplementary Figure 20: Specific heat as a function of temperature measured in zero field, $B = 0.1$ T and 1 T. The inset shows zero field data in the low temperature regime in more detail.
